# Embedded Sensors for Structural Health Monitoring: Methodologies and Applications Review

**DOI:** 10.3390/s22218320

**Published:** 2022-10-30

**Authors:** Pedro M. Ferreira, Miguel A. Machado, Marta S. Carvalho, Catarina Vidal

**Affiliations:** 1 UNIDEMI, Department of Mechanical and Industrial Engineering, NOVA School of Science and Technology, Universidade Nova de Lisboa, 2829-516 Caparica, Portugal; 2 Laboratório Associado de Sistemas Inteligentes, LASI, 4800-058 Guimarães, Portugal

**Keywords:** embedded sensors, sensing technology, smart materials, structural health monitoring, non-destructive evaluation

## Abstract

Sensing Technology (ST) plays a key role in Structural Health-Monitoring (SHM) systems. ST focuses on developing sensors, sensory systems, or smart materials that monitor a wide variety of materials’ properties aiming to create smart structures and smart materials, using Embedded Sensors (ESs), and enabling continuous and permanent measurements of their structural integrity. The integration of ESs is limited to the processing technology used to embed the sensor due to its high-temperature sensitivity and the possibility of damage during its insertion into the structure. In addition, the technological process selection is dependent on the base material’s composition, which comprises either metallic or composite parts. The selection of smart sensors or the technology underlying them is fundamental to the monitoring mode. This paper presents a critical review of the fundaments and applications of sensing technologies for SHM systems employing ESs, focusing on their actual developments and innovation, as well as analysing the challenges that these technologies present, in order to build a path that allows for a connected world through distributed measurement systems.

## 1. Introduction

The design, fabrication, construction, and implementation of Embedded Sensors (ESs), smart materials, and smart structures are currently among the greatest challenges in engineering research; additionally, innovation regarding sensors and sensor systems are essential for the development of smart structures technology [1]. In this regard, Structural Health Monitoring (SHM) consists of monitoring structures and structural components in real-time and throughout their life cycle, including during their manufacturing process, without compromising their structural integrity. Structural health should remain as specified during the design stage, although it can be changed due to normal ageing and use, environmental action, and accidental events. The concept of SHM can be tackled from a periodic-monitoring perspective, through periodic maintenance actions, or from a continuous-monitoring perspective, using Sensing Technology (ST), such as embedded sensors and smart materials. Fibre Optic Sensors (FOSs) and Piezoelectric Sensors (PSs) are some of the most widely used technologies for the development of these types of materials, although there are other technologies, such as capacitive methods, electromagnetic techniques, and materials with characteristics and/or properties that can be used for structural monitoring, such as Shape Memory Alloys (SMA), as will be verified throughout this review.

Currently, continuous and real-time SHM systems are assisted with the classical Non-Destructive Testing (NDT) techniques, such as ultrasounds [2], X-rays [3], infrared thermography [4], holographic interferometry [5], eddy currents [6,7], and terahertz [8], among others, which require highly specialised labour along with expensive procedures. Furthermore, periodic inspections, which are the most traditional form of structural monitoring, are unable to provide any information on accidents and failures that occur between two successive revisions. Consequently, there is a growing interest in the development of sensitive materials or structures that integrate sensors that provide real-time information about the material itself or its environment. The use of these sensitive materials offers a good opportunity to implement health-monitoring systems that can operate throughout a component’s life cycle. The continuous monitoring of the material’s integrity will result in its greater durability and reliability. ESs must satisfy a set of requirements, i.e., they must not damage the structure, they must achieve similar conventional NDT techniques’ sensitivity, and be able to monitor a significant part of the structure [9].

ST has been in constant development, resulting in successful applications, but there are a set of challenges that motivate research and development in this area, such as new sensors that can find the exact location of the damage and its characteristics, or that can monitor structural resilience, for example. Moreover, it is essential to ensure the long life of ST, or, alternatively, to create sensors that are easily replaceable. Wireless sensor technology represents a step forward since the use of wired sensors causes many problems, including the increased cost of applications and the cost of labour, as well as reducing the reliability of data transmission [10]. With the development that is underway in nanotechnology, it is important to invest resources in sensors inspired by this area, thus guaranteeing the possibility of implementing sensory networks in topologies and variable structures. The implementation of these sensors translates into an increase in the reliability in detecting structural damage. On the other hand, more sensors will generate more data, so it is necessary to develop models for data analysis and processing for storage while simultaneously ensuring their efficiency [11,12].

Since ESs are developed and optimised for monitoring certain physical and mechanical properties in specific structures and performance under particular conditions, in this article, an in-depth review is carried out regarding the state of the art of their development and innovation. The typologies of ESs that currently exist are presented, as well as the fundamentals and physical principles underlying this technology and its applications. A comparison is made among ESs, highlighting their advantages and disadvantages, which concludes with the exposure of the challenges that this technology presents for the near future.

## 2. Fundaments and Techniques of SHM

SHM systems are developed based on a set of elements that represent the essence of implementing these systems. First, an SHM system consists of a sensory network connected to the structure or structural component, a network that can in turn consist of a set of integrated sensors and possibly smart materials as well. This is the main difference when compared to conventional NDT techniques. The sensory network is essential for conducting automated and continuous inspections, but the high number of sensors running continuously generates a large number of data that need to be processed, and in many cases in real-time. Therefore, it is necessary to have optimised data-processing facilities to ensure the instant analysis of structures or structural components through the instantaneous acquisition of monitoring data. Finally, there are essential algorithms that analyse the stored data, with appropriate corrections for environmental factors, to predict the location of the damage and its characterization [13,14,15].

There are a set of monitoring techniques that are currently used in the widest applications, which are classified as vibration-based techniques [16], FOSs [17], PSs [18], electrical resistance techniques [19], electromagnetic techniques [9], eddy current techniques [20,21,22], and capacitive methods [19].

Vibration-based techniques, also known as modal analysis techniques, which analyse the dynamic response of a structure or structural component when excited by a spectrum of frequencies, are the most widely used type for civil engineering applications [23,24], such as wooden and composite structures [16,19]. Additionally, these techniques can also be implemented in the analysis of structures subject to environmental factors [25], contact detection, and force sensing [26].

The implementation of technology with FOSs consists of an instrument capable of transforming a certain physical or chemical parameter into information to be monitored by varying parameters that define the optical wave, such as intensity, phase, wavelength, and polarisation. Based on the type of parameters that are changed, different types of optical sensors have been developed, in which the most used are the intensity-based sensors, the phase-modulated optical fibre sensors or interferometers, and the wavelength-based sensors or Fibre Bragg Gratings (FBG) [9,27,28]. FOSs offer great potential for SHM applications. Their importance for structural integrity-monitoring applications stems from factors such as their long-life cycle, high-temperature resistance, flexibility, immunity to electromagnetic interference, and reduced implementation costs [29,30]. In this regard, their application becomes very versatile and can be used for monitoring buildings, bridges, highways, pipelines, tunnels, dams [30], and railways [31], or for fire safety studies and structural fire applications [32], composite materials [33], or wooden structures [19].

Traditional non-destructive ultrasonic inspection techniques suffer from challenges such as acoustic coupling, structural accessibility, and a low signal-to-noise ratio in highly attenuating materials. The use of embedded or attached PSs overcomes some of these difficulties as they remain permanently attached or embedded in the structure or structural component throughout its life cycle, including during its manufacturing process. So far, most inspections using ultrasonic emission techniques have focused on piezoelectric transducers. The most significant design techniques based on piezoelectric transducers can be classified into three classes, as their behaviour can be passive, active, or mixed. These main classes are acoustic, acoustic-ultrasonic emissions using piezoelectric transducers, and electromechanical impedance. The latter is one of the most promising techniques for the development of SHM systems because it is very simple to implement and uses low-cost, small, and lightweight PSs. It should be noted that PSs have a wide range of applications and can be implemented in metallic or composite structures [7,9,34,35,36,37,38,39].

The electrical resistance techniques can use a particular material or structural component as a sensitive material, i.e., this technique is based on the variation of the resistance of a given material. An example of the application of this technique is the monitoring of carbon fibre-reinforced polymers (CFRP), since carbon fibres are electrical conductors incorporated in an insulating matrix. The measurement of global electrical resistance appears to be a valuable technique for monitoring fibre cracking in unidirectional arrangements as well as in the delamination process. Therefore, carbon sensors can be used for the in situ monitoring of the structural integrity of industrial composite components (primary structures), such as aircraft wings and helicopter blades, in real-time, and possibly with lower costs when compared to current composite structure inspection techniques. Nevertheless, much has yet to be achieved in this area [40,41,42,43].

Inspections by eddy currents are one of the NDT techniques that are based on the principle of electromagnetism. The electric current of a coil creates the primary magnetic field, which in the presence of a conductive material, induces alternating electrical currents in the component. Consequently, these create a secondary magnetic field, contrary to the primary field, which is measured using another coil. Induced currents circulate in planes perpendicular to the magnetic flux, usually parallel to the coil winding [44]. Damage changes materials’ conductivity, thereby affecting eddy currents and modifying the secondary magnetic field. These techniques can be used to measure electrical conductivities and magnetic permeabilities, detect defects, detect and analyse corrosion in the material, and measure coating thicknesses [6,7,20,21,45].

Other techniques that make use of the component’s electrical properties are the low-frequency electromagnetic techniques, which monitor the integrity of a given component by measuring the electrical conductivity and dielectric signature of the components [42,43], as well as capacitive methods, in which electrodes are placed on the outer surface of the sample and electric tension is applied between them, creating a condenser system, wherein capacity changes are indicative of internal properties (such as the materials’ nature or their humidity content) [9,19]. In addition, continuous wave terahertz imaging was found to be especially interesting for imaging water infiltrations and composite materials that contain conductive wires [8]. Thermography techniques have also been used to monitor the health of systems, such as the innovative variant of active transient thermography known as double active transient thermography, which increased the temperature contrast for delamination defects at different depths and locations [4].

Currently, there is a very wide set of techniques that allow for the accomplishment of effective and functional SHM systems. One of the main elements of SHM is the sensory network, which is a set of sensors placed strategically on the surface or inside of the structural component, or even smart materials with sensory characteristics, networked in a fashion that allows for the permanent monitorization of structural components or structures. ESs comprise a developing field, and in the last years, studies have been intensified because the challenges that ST imposes are incentives for the progression of science. Consequentially, it will be possible to learn more about the applications and the challenges that ES technology currently provides.

## 3. Sensor Technology

ST focuses on the development of sensors, sensory systems, or smart materials that detect a wide variety of the properties of structural components. Today’s technology already enables the monitorisation and detection of stimuli that exist around us, using extremely accurate sensory systems, which are inexpensive to install and maintain and energy-efficient [46]. Therefore, it is fair to say that sensors are vital components for creating value in existing technological processes and their industries.

**Figure 1:**
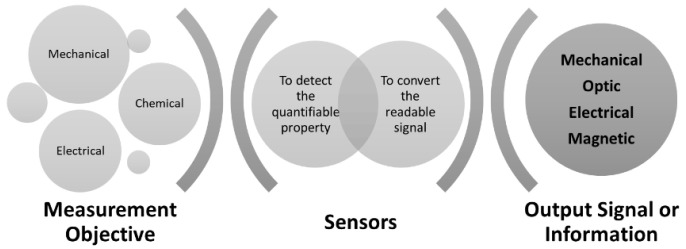
Sensor’s definition.

Sensors are technological devices that enable the quantification of the physical, chemical, or biological properties of materials, converting them into signals measured by appropriate equipment, as illustrated in Figure 1, and a wide variety of existing sensors are available for any industrial application. In addition, for more demanding industrial applications, sensors can help to improve processes and offer significant protection for industrial equipment or components [30,31].

The existence of sensors in industrial and structural components enables the detection of defects or damage and the acquirement of reports on structural integrity. Data from the implementation of sensors are processed and analysed by a set of instruments and algorithms for data analysis, and if any anomalies are identified, a set of preventative and monitoring actions is carried out to ensure the safety of industrial components [1,30,31].

**Figure 2:**
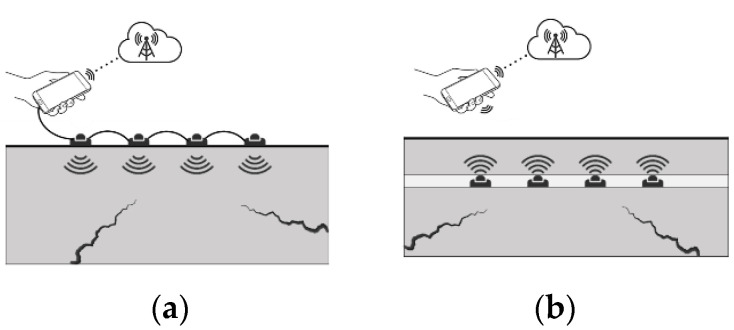
Implementation of monitoring sensors: (**a**) surface sensors and (**b**) embedded sensors.

With the constant evolution of ST and its recent developments, it is important to point out that sensors can be divided into Surface Sensors (SSs) and Embedded Sensors (ESs). The difference between them is depicted in Figure 2. The SSs are applied and coupled to the surface of components, thereby enabling life cycle monitoring. However, they are susceptible to damage from environmental factors or service conditions, including during the manufacturing process. The ESs are integrated into components, which can result in smart materials or smart components that can monitor themselves during their life cycle and the manufacturing process.

**Figure 3:**
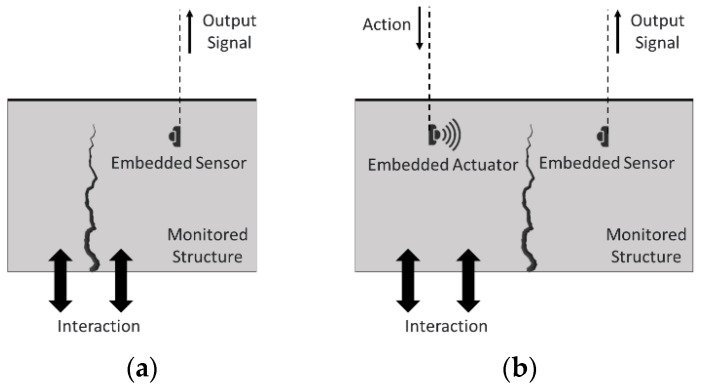
Component-monitoring approaches with embedded sensors: (**a**) passive and (**b**) active monitoring.

ST can lead to two monitoring methodologies, i.e., depending on how the sensors are implemented, either passive or active monitoring. Figure 3 illustrates the two possible approaches to monitoring a component. In passive monitoring, the information for the analysis comes from the variation of the component’s physical properties under inspection, a variation that is caused by interactions that the component suffers throughout its life cycle. This type of monitoring requires that the components under inspection have certain physical properties, such as piezoelectricity, pyroelectricity, and thermoelectricity, among others [9,47,48,49].

In active monitoring, the information for analysis comes from the application of stimuli from an embedded actuator. The capture of the response caused by stimulus is achieved by a set of sensors, embedded or on the surface. This type of monitoring requires that the components to be inspected have certain physical properties, such as piezo-resistivity, pyro-resistivity, and thermos-resistivity, among others [9,47,48,49].

SSs are the most conventional sensors used in structural integrity-monitoring applications and are based on the transmission of electrical signals. However, they easily suffer electrical or magnetic interference; therefore, in the last 20 years, intense developments in the field of FOSs have been achieved. FOSs provide a more beneficial alternative for the inspection of SHM systems and future smart structures compared to traditional technologies.

Currently, the ESs are under intense development. Review studies, such as those of Wang et al. [50] and Janeliukstis et al. [51], have already been conducted. Wang et al. [50] investigated the incorporation of thin-film piezoelectric sensors within aircraft composite components. This monitoring technology is quite versatile. However, other technologies may provide better results, as will be seen throughout this work. Moreover, the monitoring of metallic parts in aircrafts is very important, since they are the main material in these applications; however, they are not included in the Wang et al. [50] analysis. Relative to the work of Janeliukstis et al. [51], a larger analysis was carried out, reviewing and presenting the limitations of technologies with respect to incorporating piezoelectric sensors and fibre optic sensors in composite components.

This study, on the other hand, provides an overview of the existing sensor technologies that can be embedded—as well as the processes and embedding techniques available and their associated limitations—in metallic and composite components. With the progress of science, new mechanisms arise from the development of systems integrated into structural components, in addition to the advancement of smart materials, which is increasingly close to obtaining smart structural components. For this reason, we present a section exposing the recent research developed in the ESs field.

## 4. Embedded Sensors and Its Applications

The ESs in structural components have been a topic of research in the last decades and they have proven themselves to be a dominant technology. FOSs and PSs are among the most widely used technologies for the development of ESs, although there are other technologies. Throughout this section, an overview of the state of the art is presented, namely, the technologies developed in the ESs field, their methodologies for sensor integration, and their applications.

### 4.1. Fibre Optic Sensors

FOSs have recently emerged as a promising technology for incorporation into structures or structural components. With built-in sensors, it is possible to monitor structural parameters in critical locations that are not accessible for traditional sensors. In addition, these sensors can be used to validate or improve a project during the design stage or to obtain information about the performance and structural integrity of the in-service components.

FOSs are made of long-lasting materials (e.g., silica), which are resistant to corrosion and high tensile loads, and possess elongations up to 5%, leading to long life cycles. These sensors’ resistance to high temperatures enables the measurement of temperatures from 200 to 800 °C with a silica core, and up to 1500 °C with a sapphire core, wherein the measurement resolutions are on the order of 0.1 °C. Another important feature is the flexibility that these sensors have because they can be applied to complex surfaces that are difficult to access, as well as perform local or distributed measurements, which can range from 1 mm to tens of kilometres [29,30].

Each type of FOSs is based on a set of principles underlying the propagation of the optical wave and its physical properties. Although optical fibres are present at their base, FOSs can undergo geometrical (size and shape) and optical changes (refractive index and mode conversion) due to various environmental disturbances, while transmitting light from one place to another. These phenomena are unwanted; thus, over the years, attempts have been mase to minimize such adverse influences in order to obtain smoother and more reliable transmitted signals. However, optical fibres have found applications in ST applications due to these optical changes that can be used to measure external stimuli. Developments in this area have shown that sensitive disturbances in temperature, voltage, rotation, and electrical and magnetic currents can be converted or encoded into corresponding changes, such as amplitude (intensity), phase, frequency, wavelength, and polarisation, in the optical properties of transmitted light [17,25,30,52]. Table 1 presents a summary of the different types of FOSs, as well as the technologies implemented, the measurements they perform, the optical wave parameters that are influenced, and an illustrative scheme of the technology [53,54,55,56].

Many intensity-based sensors, as is the case of interferometric FOSs, are local sensors that enable the measurement of changes at specified locations in a structure. Interferometric FOSs are by far the most used local sensors because they offer the best sensitivity. This measuring technique is mainly based on the design of optical changes induced in light as it propagates along the optical fibre. The light from a source is equally divided into two fibre-guided paths: one reference path and one analysis path. In the interferometric sensors, two mirrors are used that are adjusted to mix the wave and form a “fringe pattern”, which is directly related to the difference in the phase of optical waves caused by the two mirrors. The most common configurations of interferometric sensors are the FOSs Mach–Zehnder, Michelson, and Fabry–Perot [30,53,57,58].

A Bragg grating is a permanent periodic modulation of the refractive index in the core of a single-mode optical fibre. The FBG sensor, which can be easily multiplexed to measure voltages in many locations, is a type of Quasi-distributed sensor, i.e., a type of distributed Bragg reflector built into a short fibre optic segment, which reflects certain wavelengths of light and transmits all others. This is achieved by creating a periodic variation in the refractive index of the fibre core, which generates a specific dielectric wavelength mirror. Any change in the local voltage or temperature alters the core refraction index and the wave period, followed by changes in the wavelength of reflected light, which can be monitored. There are several important concerns in FBGs’ selection and associated monitoring systems. For example, the spectral overlap of the grating changes the adjacent desirable wavelength. On the other hand, side bands at the measured wavelength, the detector filter, and an inadequate light source also introduce errors into the system [17,52,59,60].

The distributed FOSs are best-suited for large structural applications since all fibre optic segments act as sensors; therefore, disturbances within various segments of the structure can be measured. This type of sensor is based on the modulation of light intensity; therefore, fractures or local damage in a structure cause variation in light intensity. Two major distributed sensor methodologies are Optical Time Domain Reflectometry (OTDR) and Brillouin dispersion. OTDR, Rayleigh, and Fresnel dispersions are used to monitor structural disturbances. On the other hand, Brillouin dispersion shows the doppler change in the light frequency that is related to the measurements. Distributed sensors have not yet found extensive use in civil structural applications due to their insufficient resolutions, weak signals, and heavy demodulation systems. However, they have great potential in civil engineering due to their inherent distributive nature, so long as their obstacles are overcome [30,53,61].

Recently, there have been scientific reports about the inclusion of FOSs in composites and certain metallic components, particularly those having a low melting point. The techniques for the inclusion of FOSs reported so far involve complex methodologies, so it is of scientific interest to look for easier ways to incorporate FOSs in these types of structures. Therefore, Section 4.1.1 and Section 4.1.2 present a set of applications and methodologies that have been developed in recent years to incorporate the sensors and ensure the monitoring of the integrity of metal and composite structural components.

#### 4.1.1. Applications for Composite Components

The components obtained with composite materials have great relevance in engineering applications, so they have become fundamental mechanisms for the control and monitoring of a component’s integrity. When referring to the SHM mechanism of composite components, this includes the real-time monitoring of the manufacturing and curing processes of composites and the in situ non-destructive evaluation of in-service structural components. So, it is difficult to perform using conventional NDT methods, thus giving rise to the possibility of using FOSs embedded in the composite component’s matrix. The use of these types of sensors has a set of advantages due to their flexibility to easily form systems or sensory networks and create smart materials, enabling the continuous monitoring of the base material.

Currently, composite materials can be obtained through a wide range of products, such as metals, ceramics, or even polymers. However, most applications of embedded FOSs focus on polymer matrix composites [62,63,64,65,66,67,68,69,70]. In the composite production phase, FOSs can be embedded in the matrix or between the laminates of the composite to monitor certain conditions, such as the composite-stacking sequence, the resin flow during processing [62], the curing process of the laminates [71], or the misalignment of the fibres, which can lead to a significant reduction in the mechanical strength of the laminates [66]. In addition, embedded FOSs can be used to monitor the residual strains and temperature profiles developed during fabrication [67]. During the post-production phase, these sensors allow for the simultaneous monitoring of strains and temperatures to which the component is subjected during its life cycle, and, in special cases, they can also be used to detect acoustic waves [70]. During the production process, misalignment, gaps, or overlaps of the laminates or fibres may arise. Such defects may endanger the component’s integrity when it is in service; therefore, the use of embedded FOSs ensures great control over the possible spread of defects during the component’s life cycle [67].

In most applications of the monitoring of composite components, sensors such as Extrinsic Fabry–Perot Interferometers (EFPI) and FBG are implemented since these types of sensors can be easily distributed throughout a real structure with a single fibre. In addition, FBG sensors enable the identification of strains on dynamic requests, while extrinsic Fabry–Perot interferometers enable the identification of transient events [70]. The EFPI and FBG sensors are embedded in polymeric matrices and used to monitor—in real-time and simultaneously—the curing process of laminate composites, ensuring effective damage detection during the composites’ manufacturing process [71]. In addition, depending on the type of defect in terms of size and materials, three significant changes in the wavelength profiles of the FOSs sensors can be observed. These changes include the shape of the wavelength profiles, changes in the length of the corresponding waves, and the wavelength profiles’ inclination during the cooling process at room temperature [66].

Considering the materials used to manufacture composites and the manufacturing process, the type of fibre and the selected orientations strongly influence the temperature profiles obtained and the residual strains. In addition, FBG sensors can accurately determine the residual strains induced during the manufacturing and post-processing stages, as well as the thermal expansion behaviour of continuous fibre-reinforced thermoplastic composites when manufactured by fused filament fabrication [67].

Different applications can make use of this type of sensor. Table 2 and Table 3 present an overview of the state of the art and the developments made regarding embedded sensors, additionally presenting the different types of FOSs used and the methodologies of the integration of sensors for each of the applications developed.

The studies carried out with respect to the monitoring of composites’ manufacture with embedded FOSs, namely, the Fibre Bragg grating and the Extrinsic Fabry–Perot Interferometer (EFPI), have shown their capability and potential in certain future applications and serve as basic knowledge towards this goal. During the process of integrating FOSs into the polymer matrix, many authors [62,63,64] reported challenges regarding sensor fastening or FOSs breaks. These problems are critical and may lead to incorrect monitoring and consequently jeopardise the component integrity analysis, requiring the implementation of mechanisms or techniques for their prevention. To solve these issues, FOSs complemented with textile reinforcements have been implemented and studied by Bremer et al. [65] and Alwis et al. [72]. This textile reinforcement will make use of its conventional counterparts, and civil infrastructure will be fully incorporated with sensors to ensure safety, comfort, and long-term durability.

Accordingly to Kuang et al. [62] and Ramly et al. [64], the composite-manufacturing process can cause the appearance of residual strain in FOSs. Therefore, it is essential to perform a predicted analysis of the residual strain because this strain may lead to deviations from the results and influence the structural component’s monitoring. In this regard, the signal obtained when FBG sensors are properly embedded and readable have a difference of minus 1 nm when compared to the signal obtained before FBG sensors were embedded in the composite matrix [64]. Therefore, the signal reduction obtained is not very significant when compared to the typical strain sensitivity of FBG sensors, which corresponds, for example, to 1.2 pm/με for the wavelength of 1550 nm, leading to the conclusion that incorporating FOSs is feasible and may be an alternative to conventional NDTs. Embedded optical fibre FBG sensors can also detect small delamination or disengagements between the matrix and fibres via an FBG spectrum change, allowing for the prediction of a fibre-reinforced polymer beam’s structural failure [69].

Regarding the correlation between FBG sensors and EFPI, each has a preferred application, which is why these types of FOS are used simultaneously, complementing the monitoring process, as shown in the works of Leng et al. [71] and Oliveira et al. [70]. For example, the curing process and bending tests can be monitored with the incorporation of these sensor types, which is extremely advantageous, since, regardless of the loading type or life phase of the structural component, mixed and completed monitoring are guaranteed.

**Figure 4:**
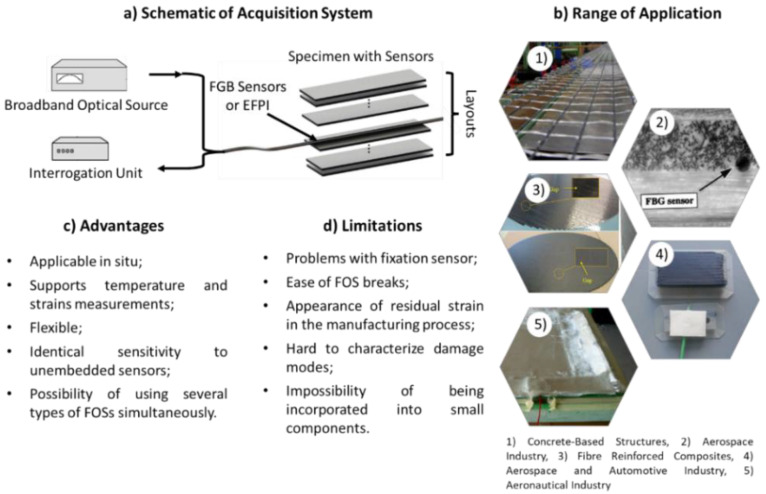
Fibre optic sensors embedded in composite components.

The main concepts related to the integration of FOSs into composite components are summarised in Figure 4. Figure 4 depicts a schematic of an acquisition system used in this technology, and a summary of the advantages, limitations, and range of applications for the incorporation of FOSs into composite components.

#### 4.1.2. Applications for Metal Components

FOSs are attractive for in situ structural monitoring, especially metallic structural components since the sensors that use optical properties provide silent monitoring, greater sensitivity, good accuracy, and high-temperature capacity.

Metals such as steel, nickel, iron, and titanium have high melting points. In this sense, metal-processing technologies involving the melting of metals will lead to the destruction of FOSs, which is undesirable. Therefore, to avoid the damage of FOSs, it is necessary to resort to a set of material-processing technologies that does not involve the fusion of a base metal, such as shape deposition manufacturing, Layered Manufacturing, Laser Deposited, ultrasonic additive manufacturing, electron beam melting, Magnetron Sputtering, and Electroplating.

Over the last few years and based on the techniques of the incorporation of FOSs, a set of systems has been developed that enables the monitoring of strains, temperature variations, and cracking using mainly FOSs of the FBG type [73,74,75,76,77,78,79,80,81,82]. Xiao Chun Li et al. [73,74,75,76] are among the main boosters in the development of methodologies capable of integrating sensors into metal components, in this case using low-temperature processes, magnetron sputtering, and electroplating.

The FBG sensors incorporated into components manufactured, for example, with nickel and stainless steel provide high sensitivity, good accuracy, and high-temperature capacity for temperature measurements. Regarding sensitivity in temperature measurements, embedded FBG sensors have better results than those shown when sensors are not embedded [75] and an accuracy of about 2 °C [74].

For strain measurements, embedded sensors in metal were capable of high sensitivity, precision, and linearity, while unembedded FBG sensors achieved similar results [75]. In addition, the results obtained by Schomer et al. [78] showed that the embedded FBG sensors accurately track the strain for temperatures above 400 °C.

The different applications that can use this type of sensor are presented in Table 4 through an overview of the state of the art and the developments made regarding FOSs. The different types of FOSs used and the methodologies of the integration of sensors for each of the applications developed for metallic components are also presented.

Xiao Chun Li et al. [73,76] have contributed to the development of methodologies for the integration of FOSs into metal components, focusing mainly on the structural components obtained via nickel and stainless steel, and not covering the components fabricated from aluminium alloys, which are currently one of the main applications. The technology developed by these authors has shown very promising results, mainly due to the integration process of FOSs developed that leads to temperature and strain measurements with satisfactory sensitivity when compared to the same unembedded sensors. Their work enables the monitoring of the residual strain coming from the manufacturing process and high temperatures but neglects the monitoring of cracks and porosity in structural components.

Alemohammad et al. [77] used a similar FOS-embedded process, incorporating the FOSs into a cutting tool and reporting results on the validation of this methodology when the component is subjected to thermal cycles, wherein said results were good and relevant. However, this research could also have focused on the analysis of strain cycles since this type of application is subjected to very high stresses that can lead to the fracture of the cutting tool.

Schomer et al. [78], Chilelli et al. [81], and Hehr et al. [82] demonstrated the feasibility of integrating FOSs into metal matrices through UAM and monitoring the temperature, cracks, and residual stress, respectively, in structural components. This type of process also has great potential for monitoring the components present in environments subject to high temperatures, which does not happen with piezoelectric sensors as will be analysed later on.

Grandal et al. [79] and Jinachandran et al. [80] implemented different methodologies to incorporate FOSs with very promising results. The sensors presented identical thermal and strain sensitivities when compared with the same unembedded sensors. These methodologies also ensure the durability, detachability, and reusability of the monitoring equipment. However, the application range is still too small, requiring expansion for application with other metallic materials.

**Figure 5:**
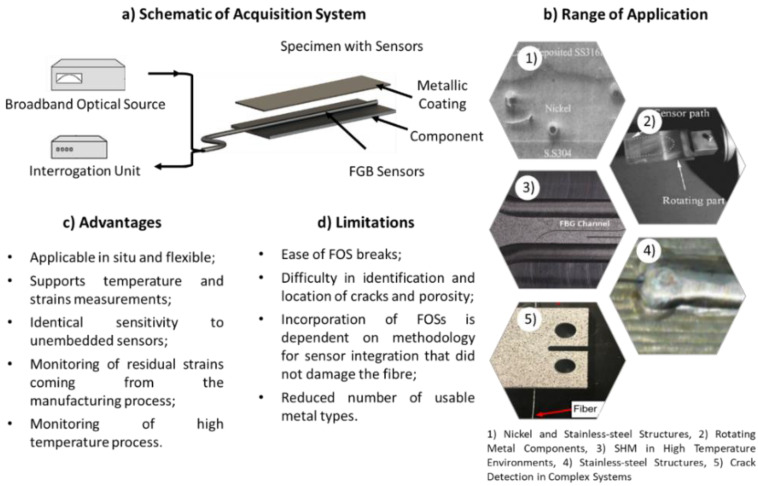
Fibre optic-embedded sensors for metal components.

Figure 5 contains summary of the current state of the art, presenting a schematic of an acquisition system, a set of advantages, limitations, and a range of applications for the incorporation of FOSs into metal components.

### 4.2. Piezoelectric Sensors

The piezoelectric effect was discovered in 1880 by the Curie brothers and was first used by Paul Langevin in the development of ultrasounds, based on quartz crystal transducers, during the first World War. The development of piezoelectric ceramics, such as Lead Zirconate Titanate (PZT) and Barium Titanate, was revolutionary. Moreover, to obtain better properties than crystals after being polarised, they also offered flexible geometries and dimensions because they could be manufactured through sintering. Currently, piezoelectric ceramics of the PZT type, in their various applications, are the predominant ceramics in the market. In addition, other materials can also be found, such as PT (PbTiO_3_) and PMN (Pb (Mg_1_/3 Nb_2_/3) O_3_), that are used in devices that require special and very specific properties, such as high-temperature transducers. However, there are even more materials that have a piezoelectric effect, which can be classified into one of the following groups: piezoelectric ceramics, quartz crystals, piezoelectric composites, hydro soluble crystals, piezoelectric monocrystals, piezoelectric semiconductors, or piezoelectric polymers [83,84].

The knowledge and electromechanical behaviour of these materials are fundamental for the industry, especially those that depend and focus on the ultrasound aspect. From the groups defined above, piezoelectric ceramics are the ones with a greater flexibility of shape and properties, being widely used in the production of ultrasound equipment, NDT, and actuators [85].

Of all these possible applications, the possibility of developing technology that allows for inspections of structural components, and the periodic or continuous monitoring of structural integrity, through traditional or innovative NDT equipment represents one of the most important applications. The most significant defect or damage inspection techniques based on piezoelectric transducers can be grouped into three classes, wherein their behaviours can be passive, active, or mixed. These main classes are acoustic emissions, acoustic–ultrasonic emissions using piezoelectric transducers, and electromechanical impedance [9].

The technique based on electromechanical impedance (EMI) is considered one of the most promising methods for the development of SHM systems. This technique is simple to implement and uses small and inexpensive piezoelectric sensors. However, practical problems have made it difficult to apply this technique to real-world structures, and the effects of temperature have been cited in the literature as critical problems [18,39].

Regarding non-destructive ultrasonic inspection techniques, there are problems regarding the reproducibility of the acoustic coupling, accessibility to the structure, and the weak signal-to-noise ratio in highly attenuating materials. The use of built-in or connected piezoelectric sensors overcomes some of these difficulties because they remain permanently connected to the structure, and these sensors can be used to monitor the integrity of a given component from its manufacturing phase to the end of its life cycle. At present, most works dealing with acoustic and ultrasonic processes have used piezoelectric transducers [9,83,84,86,87].

Recently, there have been reports in the scientific community of the incorporation of piezoelectric sensors into composites and some metals. The techniques for the inclusion of piezoelectric sensors reported so far involve complex methodologies, so it is a scientific interest to look for easier ways to incorporate piezoelectric sensors into metal or composite structures. Therefore, Section 4.2.1 and Section 4.2.2 present a set of applications and methodologies that have been developed in recent years as a way to incorporate the sensors and ensure the monitoring of the integrity of metal and composite structural components.

#### 4.2.1. Applications for Composite Components

The interest in the concept of self-monitoring structural components has grown in recent years due to its potential to enable the continuous monitoring of the next generation of smart structures [88,89,90,91,92,93,94,95,96,97,98,99,100,101]. Considering the studies developed, the applications of piezoelectric-embedded sensors for composite components are mainly based on structures or components of reinforced concrete [88,93,95,96,97,98,99]. The development of structures or components of reinforced concrete with PSs incorporated to obtain smart structures is particularly suitable for implementation in numerous fields, mainly in civil engineering, because PSs are characterized by being reliable and stable over a long term. Additionally, the use of PSs embedded in reinforced concrete structures is a unique opportunity for the SHM of civil structures due to PSs’ compatibility with new or existing infrastructures.

For this type of application, PSs based on PZT, as discs or fibres, can be used. In certain applications, a self-sensing structural material with piezoresistive characteristics, based on the addition of carbon nanotubes, can be obtained [93].

Sensors are usually attached to the reinforcing bars on key parts of reinforced concrete structures, which are susceptible to damage or are difficult to access, such as bridge shoes, pavement connections, or docks. In these applications, there are a set of parameters to be monitored, including internal strains [88]; the effect of corrosion [95]; measurements of healthy and damaged steel bars in reinforced concrete beams [96]; strengths/behaviours evaluated by in-plane tensile, in-plane tension–tension fatigue, and short beam strength tests [89]; and deformations, strain, and damage [93].

The ability to monitor structural integrity is only possible if the use of ESs is successful. In this regard, studies have shown that the actuators and PSs are sensitive to the damage in concrete and steel reinforcement bars; however, the sensitivity of the transducers depends greatly on the frequency of the excitation selected. Thus, the use of these sensors to monitor, detect concrete fissures, and analyse steel yield can be considered as a highly promising and non-destructive structural monitoring method [96].

Embedded piezoelectric sensors can also be used to monitor other types of composite materials, among which are materials obtained by glass fibre/epoxy composite laminates. In addition, it is also possible to use ESs to improve the internal stresses of the structural component. In this regard, the use of Piezoelectric Fibre Composite Sensors (PFCSs) has more advantages when compared to traditional PZT ESs, because the use of PFCSs leads to a 56% reduction in the concentration of longitudinal stresses and a 38% reduction in the concentration of transverse stresses when compared with the use of PZT ESs, although none of these types of sensors affect the fatigue behaviour of the base material [89].

For static and continuous monitoring, the techniques presented show great promise for the development of smart structural materials, which can be used to monitor the integrity of engineering systems in civil, mechanical, or aerospace structures. Possible applications for the structural components in civil infrastructure include the use of sensors integrated into columns, bridge beams, and pillars; the deployment of smart mortars and smart bricks for masonry structures; or in the structural components obtained by glass fibre/epoxy composite laminates [88,93].

This type of sensor is used to obtain different applications; so, Table 5 and Table 6 present an overview of the state of the art and the developments made regarding ESs, while also presenting the different types of PSs used and the methodologies for the integration of the sensors for each of the applications developed.

Based on the studies presented in Table 5, the PSs are incorporated into reinforced concrete and on their reinforcement bars. In this respect, the PSs have great applicability due to the ease of incorporating these sensors into the reinforced concrete matrix, since before drying the concrete, the cement mortar can be easily handled. In addition, the production process of reinforced concrete structures is carried out at room temperature, which represents an advantage towards incorporating PSs, because the use of PSs is limited by their Curie temperature, which in many applications is higher than room temperature.

Authors such as Wu et al. [88,90], Karayannis et al. [96], Gopalakrishnan et al. [97], and Sha et al. [99] have developed studies on the structural components obtained in reinforced concrete, conducting experimental tests that simulate real applications, such as the de-bonding and bending tests on reinforced concrete beams, and tensile tests on reinforcement bar. Both studies showed feasibility in identifying the presence of damage, such as concrete cracking, steel bar elongation, their locations [96], and dynamic stress-sensing capability [99]. Downey et al. [93] has also shown that the use of self-sensing structural material with piezoresistive characteristics based on the addition of carbon nanotubes into a cementitious matrix can provide these monitoring indices. However, there is still great difficulty in estimating the damage and controlling the volume of collected data, since in these types of applications there is great potential for implementing multi-sensing technology, that is, to incorporate numerous PSs into the reinforced concrete matrix. Despite the multi-sensing nature of the PZT-based sensors, connecting them in series or parallel is a promising method to reduce the volume of data collected from the sensors to identify damage in a structure [97]. Environmental factors can permanently damage the integrity of a reinforced concrete structures through the corrosion of the concrete and reinforcing bars. The authors Talakokula et al. [95] and Ahmadi et al. [98] found that incorporating PSs into the concrete matrix or in strategic locations allowed for the monitoring of the corrosion process, in turn enabling preventative action and control over what occurs inside.

Regarding polymer matrix composites, there is a small range of manufacturing processes that allow for the incorporation of PSs due to the Curie temperature that limits the applicability of these sensors. Therefore, processes such as open contact-moulding processes [89] and the vacuum-assisted resin transfer moulding [94] are good alternatives since they allow PSs’ incorporation without their disuse. As for the PSs used, composite compatibility is one of the main conditions for good monitoring operations. In this regard, according to Konka et al. [89], the conventional PZT sensors seem to have low compatibility with composites; hence, the reduction in strength values is higher when compared to piezoelectric fibre composite sensors, which seem to have very high compatibility with composites. Hence, a piezoelectric fibre composite sensor would be an ideal choice as an embedded sensor when compared with PZT sensors.

In addition to conventional PZT sensors, there is another type of ST that enables the monitoring of structural components and offers an alternative to conventional sensors. One such type of technology is presented by Lin et al. [91], who demonstrates that when combined with a sophisticated data acquisition system and diagnostic software, it can dramatically reduce inspection costs, allow for more frequent maintenance periods, and reduce the appearance of catastrophic structural failures.

Finally, Takagi et al. [92] demonstrated once again the versatility of PSs through the use of piezoelectric fibres with a metal core that functions as a sensor or an actuator for effectively controlling active vibration.

**Figure 6:**
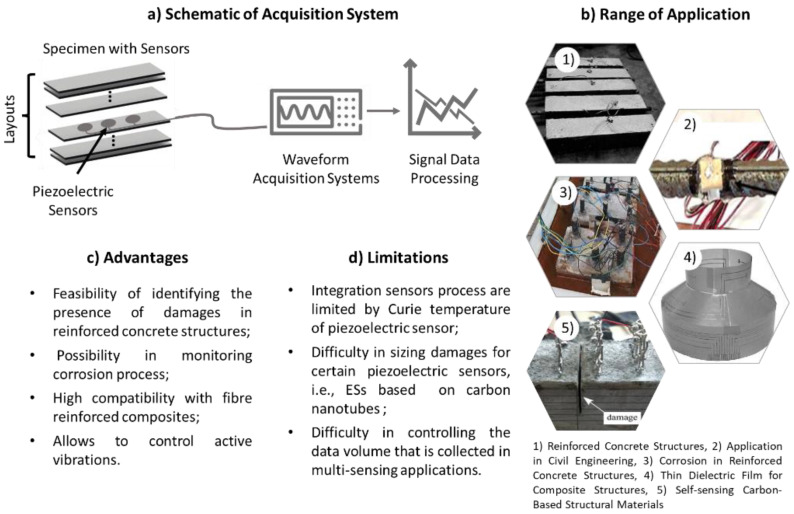
Piezoelectric sensors embedded into composite components.

The Figure 6 shows a schematic of an acquisition system used in this technology, and a review of the advantages, limitations, and range of applications for the incorporation of PSs into the composite components.

#### 4.2.2. Applications for Metal Components

Currently, the applications for metal components [91,102,103,104,105,106] use sensitive ceramic and polymeric piezoelectric sensors, more specifically PZT sensors [102] and Piezoelectric Polyvinylidene Fluoride (PVDF) sensors [106]. Traditional manufacturing approaches to incorporating active materials—such as piezoelectric materials—into metals can be problematic due to their high temperatures during production or the long curing times of the adhesives used to connect the sensor to the metal. To bridge the challenges that technological processes present, the scientific community has carried out a set of developments, among which is their focus on the development of a process of “stop and go”, which consists of taking a break in the manufacturing process of a given component to allow for the inclusion of PSs [102], or the inclusion of sensors through the joining of metal components in the solid-state, i.e., by ultrasonic additive manufacturing [106].

Based on the mechanisms that currently exist to incorporate PSs in metal components, it was possible to obtain responses of about 3 V of maximum voltage, for pressure values not exceeding 40 MPa, and good behaviours when requested with different frequencies (i.e., 10 Hz, 15 Hz, 20 Hz, and 25 Hz), for PZT piezoelectric sensors. For applications with PVDF sensors, the studies led to an average sensitivity of 9.4 mV ${\mu \varepsilon }^{-1}$ and the ability to detect strains.

The studies carried out so far proved the feasibility of manufacturing smart components with ESs. In addition, they can evaluate the components’ performance, leading to the possibility of manufacturing smart components that can have an impact in industries such as the energy, aerospace, automotive, and biomedical industries, or for applications such as air/fuel premixing, pressure pipes, and turbine blades [91,102,106].

Different applications can make use of these types of sensors; Table 7 presents an overview of the state of the art and the developments made regarding embedded sensors, also presenting the different types of piezoelectric sensors used and the methodologies for the integration of the sensors for each of the applications developed.

Based on the studies developed, the processes to incorporate PSs into metal matrices are based on the additive manufacturing process, allowing greater control of the sensors’ positioning and avoiding their damage.

The authors, whose studies are reported in Table 7, have shown that the use of PSs inside metal components allows for the monitoring of external stimuli, such as strain and temperature variations, with satisfactory sensitivities. However, there are several factors that are fundamental to be studied to validate the applicability and versatility of this strand of ST. Regarding service factors, the possibility of monitoring, detecting, locating, and sizing possible damage or cracks is essential to ensuring the integrity of structural components. With respect to environmental factors, the action of corrosion can lead to irreparable consequences in the metal structure, as it is essential to study the possibility of PSs’ ability to monitor the corrosive actions that a metal structure is subjected to during its life cycle.

**Figure 7:**
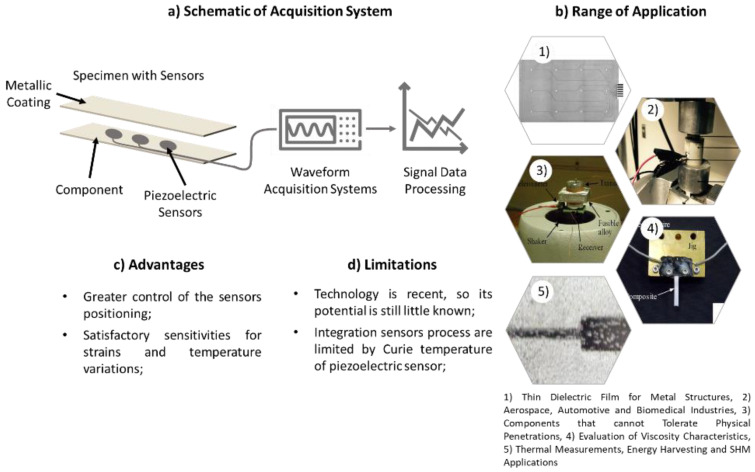
Piezoelectric sensors embedded into metal components.

In Figure 7 presents a summary of the current state of the art, presenting a schematic of an acquisition system, a set of advantages, limitations, and a range of applications for the incorporation of PSs into the metal components.

### 4.3. Other Embedded Sensors

In this regard, a set of applications that use sensors or sensory systems with optical properties (FOSs) or with piezoelectric properties has been analysed. Although most SHM applications are based on these two technologies, it is important to note that they are not the only ones; in this sense, other structural monitoring alternatives with ESs will be analysed throughout this section. These alternatives are monitoring technologies based on SHM techniques, such as capacitive methods, electromagnetic techniques (for example, eddy currents), and materials with characteristics and properties that can be used for structural monitoring, such as Shape Memory Alloys (SMA).

Several studies have been developed using these SHM techniques to obtain self-monitored structural components, mainly for application in composites and metal components. Therefore, Section 4.3.1 and Section 4.3.2 present a set of applications and methodologies that have been developed in recent years as a methodology to incorporate sensors and ensure the monitoring of the integrity of metal and composite structural components.

#### 4.3.1. Applications for Composite Components

In the last two decades, there has been significant growth in the development of multifunctional technologies to improve materials’ properties. Multifunctional technologies enable the acquirement of SHM systems in order to detect structural damage and for strain sensing.

Regarding applications obtained in composite materials, a set of embedded SHM techniques has been developed to incorporate the structural components [107,108,109,110,111,112,113]. Starting with SHM techniques that have capacitive methods as their basis, the scientific community has developed technologies that allow for the monitoring of the deformations that a particular component is subjected due to dynamic impacts by including a pair of wires in adjacent layers to obtain a complex condenser. With the capacitance variation of this condenser, it is possible to monitor the deformations inflicted on the structural component [111]. In another approach, the authors developed a built-in monitoring sensor to monitor the water content inside reinforced concrete structures based on a passive and wireless condenser resonance circuit [107].

Regarding the electromagnetic techniques based on SHM techniques, techniques have been developed to determine the humidity content and, consequently, the deterioration of reinforced concrete structures. To this end, smart sensors are incorporated into concrete structures for real-time monitoring. These sensors are microstrip patch antennae that generate a set of electromagnetic waves allowing for the determination of the degree of humidity in the structure [110].

The development of SHM systems also employs materials that have interesting properties, as is the case of Shape Memory Alloys (SMA). SMA are metal alloys that, when deformed, return to their initial format if heated. These materials are generally lightweight, found in solid-state, and present an alternative to mechanical actuators such as hydraulics, pneumatics, and motorised systems. These alloys have applications in the robotics, automotive, aerospace, and biomedical industries. In addition, these materials can be incorporated within the traditional carbon fibre-reinforced polymer composites to increase the mechanical properties of composite panels and explore their intrinsic electrothermal properties. That is, with the variation of the electrical resistance and the internal resistance-heating source provided by the SMA network, it is possible to perform rapid monitoring of the strains’ distribution and an in situ visualization through thermographic images of the damage [108].

The methodologies for integrating this type of sensor or materials are fundamentally based on a reinforced concrete structure, on the inclusion of sensors during the production phase and in polymer matrix composites, and on the inclusion of these before the curing process. However, new approaches to methodologies for obtaining smart sensor materials are beginning to emerge, such as the use of magnetron sputter deposition to deposit thin films on heat-sensitive materials such as fibre-reinforced polymers, also known as composite materials [112].

The different applications that can make use of this type of sensor are presented in Table 8 through an overview of the state of the art and the developments made regarding ESs, also presenting the different types of ESs used and the integration methodologies of the sensors for each of the applications developed.

In addition to fibre optic and piezoelectric ESs, many authors have developed another type of ES aiming at monitoring other material properties and structures that are not possible with FOSs and PSs, namely, the group of capacitive methods. These methods enable the monitoring of the moisture content of reinforced concrete structures [107], which is a huge advantage since excess moisture leads to the appearance of biological agents, cracks, and delimitations. The monitoring of components with lattice structures is also a great challenge due to the difficulty of incorporating sensors; however, the use of capacitive methods enables researchers to overcome this difficulty and, consequently, evaluate the internal efforts of this type of material, as detailed by Ong et al. [107]. However, these types of ES have some limitations that can affect their performance, i.e., capacitive methods are much more sensitive to changes from environmental conditions, such as temperature and humidity variations, although, under certain conditions, this sensitivity can be easily changed. In addition, the measurement of capacitance or electrical resistance can be easily misinterpreted.

Other material properties can be used to inspect structural components. The use of SMA is another alternative and, according to Pinto et al. [108], it is possible to monitor, scale, and locate the appearance of a broken fibre, crack, or delamination in carbon reinforced plastic composites through thermography. At the same time, it allows for the monitoring of stress–strain behaviour due to the thermo-mechanical behaviour of SMA. However, thermography does not have a sufficient resolution to identify small defects and has difficulties in quantifying damage depth [108].

The cost, weight, or physical size of the sensors restrict the total number that a structure can accommodate—which is often the case for complex systems—and leads to an abundance of data for processing. In this regard, the use of carbon nanotube fibre sensors enables lower costs and weight and ensures a simple and easy way to incorporate the fibres inside the composite [109]. Another advantage of this application is that the use of carbon nanotube fibre sensors embedded in composites requires only a simple measurement of the electrical resistance to monitor the efforts that are applied in the component [109]. Therefore, Meoni et al. [113] applied this type of sensor inside reinforced concrete structures, leading to the development of a viable measurement technique. However, carbon nanotube technology is recent, so its true potential is still unknown, and the production process of carbon nanotubes is relatively expensive.

A procedure using ceramic PZT powders as a self-sensing composite material was also successfully developed. The piezoelectric powder is interleaved between the glass fibre-reinforced polymer prepreg plies and the piezoelectric signals are collected using brass-sheet electrodes. Fibre Bragg gratings and piezoceramics have been proposed as real-time sensors integrated into laminates. Their presence, however, has a negative impact on the mechanical properties of the hosting laminate. So, Gino et al. [114] demonstrated that PZT powder laminates have higher sensitivity than PZT commercial disk laminates and that the mechanical properties of the powder laminate are comparable to the non-sensing reference counterpart. This is a new technology that the authors of this paper believe has enormous potential.

A new breed of implanted nanocomposite sensor network has been developed for implementing an in situ, ultrasound tomography-driven SHM of carbon fibre-reinforced polymer (CFRP) laminates. Individual sensing units were formulated with graphene nanosheets using a spray deposition process, circuited with highly conductive carbon nanotube fibres as wires, and then implanted into CFRP laminates to form a dense sensor network [115]. Monitoring the moisture content inside the concrete structure is one of the most critical factors to ensure structural integrity. However, many of the types of equipment or technologies used to measure moisture content are destructive and require additional drilling in the material to perform measurements. In this regard, Teng et al. [110] developed a microstrip patch antenna that presents a precise calibration, validated by a numerical model, which is reliable, easy to use, and is implemented inside the structure. However, self-generated energy for data transmission remains a challenge for these technologies, because the power supply typically has a longer service life than the structures in which these types of sensors are integrated [110]. Wireless passive sensors can offer a good solution to these problems. Radio Frequency Identification (RFID)-passive sensors do not require batteries or maintenance so the sensors can be embedded in structures such as walls, packaging, or in clothing. Consequentially, a sensor’s lifetime must be the same as the lifetime of the structure in which it is embedded. Thus, the output from the sensor can be read through different materials [116,117]. RFID with sensing properties is predicted to become a key product of the next generation because it can also be used in force measurements since strain is proportional to force. [118]. The traditional RFID tag is typically used in power supply and data transfers [117]. One problem with this technology is the low power output of the tag. RFID-based sensing has often been limited to low power consumption sensors such as those used in temperature sensing [119]. In Suzuki et al.’s work [117], a displacement sensor was developed using an external strain gauge and two tags, one providing power for the on-board electronics and strain gauge and the other tag for transferring data. The sensor was tested in “real” conditions and the reading of signals through various materials used in buildings was successfully performed.

Embedded sensors allow for the addition of value or functionalities in structural components; however, they can compromise the structural properties of the host material. Therefore, the work developed by Cougnom et al. [112] presents an alternative that does not deteriorate the properties but rather guarantees an increase depending on the typology of the thin films deposited. Consequently, this composite material enables the fabrication of single-metal thermocouple thin and heating elements.

**Figure 8:**
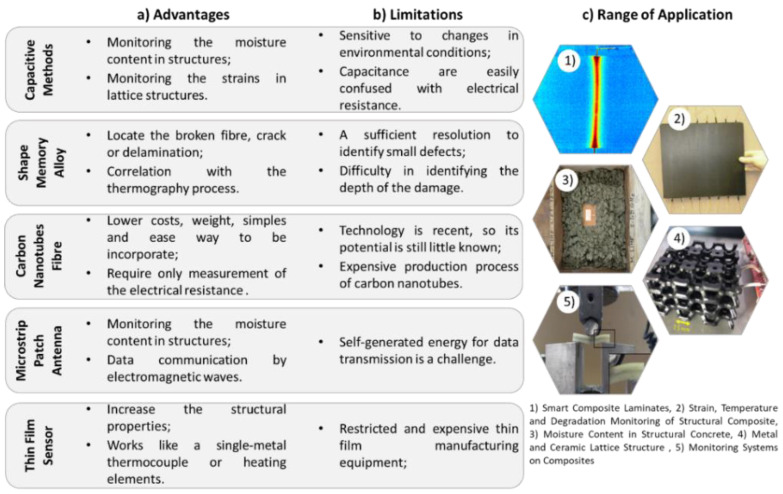
Other embedded sensors for composite components.

Therefore, Figure 8 shows the advantages, limitations, and range of applications for each of the technologies presented throughout this section. The advantages and limitations are related to the behaviour that the structure presents with respect to the type of embedded sensor used.

#### 4.3.2. Applications for Metal Components

ESs’ monitoring of components manufactured with metallic materials is a more complex process when compared to the components obtained with composite materials [73,120,121,122,123,124]. In this regard, technological processes such as laser-assisted metal deposition, low-temperature processes, magnetron sputtering and electroplating [73,121,124], ultrasonic metal welding [120,122], and a hybrid-manufactured metal process with an in situ process interruption [123] are used to incorporate sensors or materials that enable continuous monitoring.

Through the technological processes mentioned, there is the possibility of incorporating sensors or materials, such as thin-film [73,120] or shape memory alloys [122], to monitor the thermomechanical behaviour of structural components, as well as eddy current sensors [124] for studies of crack propagation and its evolution over time.

This type of sensor has been implemented in practical applications; so, Table 9 presents an overview of the state of the art and the developments made regarding ESs, also presenting the different types of ESs used and the integration methodologies of the sensors for each of the applications developed.

Li et al. [73] and Cheng et al. [120] developed technologies for temperature monitoring in metal structural components through thin-film thermos-sensors but used different methodologies for the sensors’ integration, i.e., the laser-assisted metal deposition and the ultrasonic metal-welding techniques, respectively. In the case of the application studied by Li et al. [73], it is notorious that there is a spread of strain values measures, which, according to the author, is due to the acquisition process’ limited resolution and the electrical noise generated during the amplification and transport of the signal. Therefore, it can be concluded that although the process of integrating thin-film thermal sensors has been well-achieved and presents a response to external loads, the signal obtained is very noisy and has poor resolution. Regarding the study accomplished by Cheng et al. [120], a monitoring sensitivity identical to traditional thermocouples was obtained and provided strong evidence that the heat generated during ultrasonic welding may not be critical for structural integrity. In this regard, this type of ES has great potential to improve the understanding of numerous other manufacturing processes by providing in situ monitoring with high spatial and temporal resolution in critical locations.

Still, in the context of temperature monitoring, Zhang et al. [121] developed a small sensor, i.e., a micro-photonic sensor, which allowed the authors to obtain data with a significantly improved spatial and temporal resolution and a sensitivity higher than many applications with FOSs. As the operation of this sensor is based on optical properties, they present immunity to electromagnetic interference, and they are suitable for operation and monitoring in processes with a high operating electrical voltage and/or current, such as resistance welding, work involving high-voltage cables, etc. However, the challenges of incorporating this type of micro ring sensor arise from the fact that most metal structures have a hostile manufacturing environment and require sensors to be manufactured and incorporated before they are tested in an industrial environment.

The ultrasonic additive-manufacturing process is one of the main methods that allow for the incorporation of different materials into a metal matrix. Hahnlen et al. [122] demonstrated the possibility of obtaining aluminium alloy composites with shape memory NiTi, magneto-strictive Galfenol, and electroactive PVDF phases. This enables the monitoring of properties such as the stresses and strains inside of a metallic structure, the non-contact sensing of composite stress and strain utilizing the embedded magneto-strictive material, and vibration-sensing properties, respectively.

Juhasz et al. [123] described the implementation of an internal passive sensor printed on a hybrid-manufactured metal structure during an in situ process interruption. This hybrid process combined the benefits of traditional manufacturing (machining) with additive manufacturing, resulting in more complex structures composed of several materials, with this combination being one of the main advantages of the hybrid processes. The greater benefit of the hybrid process is the potential access to internal cavities machined within an intermediate layer structure during manufacturing to place components.

Among the NDT technologies available, the eddy current technique has some advantages, such as robustness and no requirement for surface preparation or couplings [45]. In addition, they feature compact and suitable solutions for incorporation into SHM applications [124]. According to Sholl et al. [124], it was possible to develop an application that provides real-time data on the dimensions of a crack, allowing this type of sensor to be connected to a monitoring centre and consequently triggering a set of reparations or replacements according to the state of crack propagation. However, if a defect or planar crack does not cross or interfere with the current, this will not be found and may endanger the integrity of the component.

**Figure 9:**
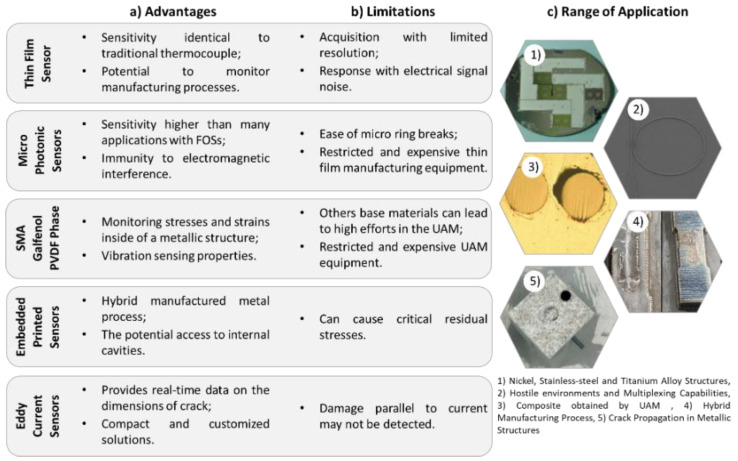
Other embedded sensors for metal components.

Figure 9 shows a review of the advantages, limitations, and range of applications for each of the technologies presented throughout this section. The advantages and limitations are related to the behaviour that the structure presents with respect to the type of embedded sensor used.

## 5. Methodology for Sensor Integration

Integrating sensors inside a given component is one of the major challenges in the development of self-monitored structures since the integrity of both the sensor and component must be ensured. In this regard, the process of integrating the sensors into the structural components is not straightforward, as these components can consist of metals or polymers, or a set of materials, as in the case of composites. The ESs used are limited to the processing technology used to embed the sensor due to their usual high temperature sensitivity and to the possibility of damage during the incorporation process. In addition, the selection of the technological process depends on the base material’s composition.

Based on the applications and technologies analysed in the previous section, it can be concluded that there are appropriate technological processes in place or methodologies for incorporating sensors into structural components. Therefore, in Section 5.1 and Section 5.2, a set of technological processes for the manufacture and processing of composites and metallic materials are presented and analysed.

### 5.1. For Composite Components

**Figure 10:**
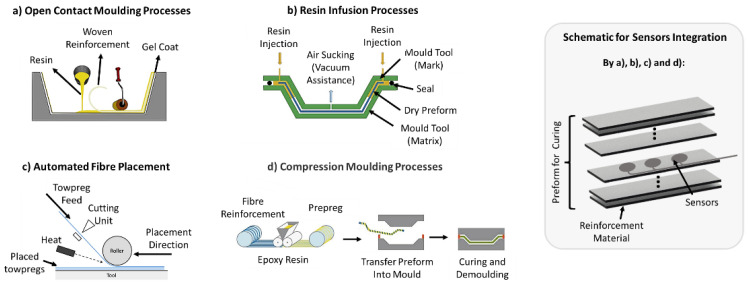
Methodology for sensor integration into composite components.

Currently, it is possible to obtain enough types of composite materials; accordingly, a set of techniques for the processing and manufacture of composite materials has been developed, which can be divided into several typologies. Among them exist open moulding (Figure 10a), resin infusion processes (Figure 10b), and high-volume moulding methods, such as automated fibre placement (Figure 10c), compression moulding (Figure 10d) [125,126,127], and spray deposition processes [115].

Based on the existing technological processes for the manufacture of composite materials, there are already studies that have experimentally validated the use of embedded sensors in composite materials. Therefore, FOSs and PSs are the main technologies used for incorporation of ESs into composite materials, mainly making use of advanced composite materials and fibre/metal laminates, carbon fibre-reinforced polymer laminates, sandwich composite panels, and continuous fibre-reinforced thermoplastic composites fabricated through the fused filament fabrication technique [62,63,64,65,66,67,68,70,71,88,89,91,92,94,95,96,97,98,99].

### 5.2. For Metal Components

Metallic structures or structural components represent a large part of the applications in engineering; therefore, the methodologies of integrating sensors into these types of components are fundamental. However, most technological processes for the manufacture and processing of metallic materials may compromise the integrity of sensors or sensory components. In this regard, it is essential to combine sensors’ physical limits with the manufacture or transformation’s technological process so that it is possible to ensure the most efficient structural monitoring possible.

**Figure 11:**
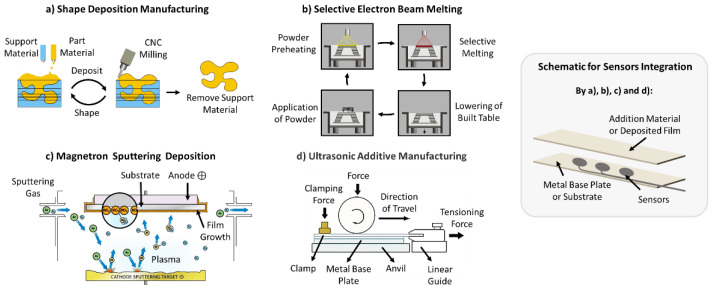
Methodology for sensor integration into metal components.

Currently, certain applications have already been validated; that is, the use of FOSs and PSs embedded in metal components are already possible through a set of manufacturing technologies with characteristics that allow for the integrity of the sensors. These technologies are the shape deposition-manufacturing (Figure 11a), the electron beam-melting (Figure 11b), the magnetron-sputtering and electroplating (Figure 11c), and the ultrasonic additive-manufacturing (Figure 11d) methods [73,74,75,76,77,78,79,81,82,102,103,105,106,120,121,122,123,124,128,129,130,131].

The sensor integration methodologies for metal components are mainly based on solid-state-processing technologies, layered manufacturing or electroplating, and laser-deposition techniques. This strand is still under development; however, there are already applications with sensors, integrated circuits, or actuators incorporated within structural structures or components, which are fully functional.

## 6. Discussion and Challenges in Embedded Sensors

Generally, all sensors or monitoring systems present a set of challenges that must be overcome to ensure high structural reliability, such as the accurate detection of damage. The use of wiring to connect sensors causes many problems, including the associated cost of its application, as well as the reduction in the reliability of data transmission [51,132,133,134]. The use of embedded FOSs is associated with a set of challenges. These are a result of the operational characteristics of the FOSs and the incorporation processes used. Regarding the incorporation of FOSs in composite components, the processes of fixing and handling FOSs are sometimes delicate situations and can lead to optical fibre breakdown; thus, not only is the insertion area of optical fibres important, but so too is the protection of the optical fibres to ensure that monitoring is not affected. Positioning the FOSs is also a challenge because the orientations of composite reinforcement fibres influence the spectrum response of the FOSs after fabrication, which can lead to insensitivity towards crack propagation. As far as the curing process is concerned, this can lead to non-uniform strains causing noisy signals and impeding the monitoring of components. Mechanical degradation due to the poor mechanical properties at the sensor–composite material interface is a common issue in these applications, especially when it comes to soft and flexible composite structures. Given the soft nature of unconsolidated textile reinforcement fabrics, the mismatch between the sensor and the fabric is a challenging issue from the perspectives of both sensor measurement and fabric properties [135,136].

Embedding the FOSs in metallic components is a more challenging process, as one of the main challenges is to ensure that the FOSs are not damaged during the incorporation phase, as some of the technological processes used require high pressures (as is the case of the UAM), and the FOSs are very fragile. In this regard, although the FOSs remain functional at higher temperatures when compared to piezoelectric sensors, it is important to develop protection and reinforcement systems for FOSs to withstand temperatures above the melting point of metallic materials to increase the range of applications. Regarding the fixation and handling process, the challenges are like those presented for composite components. The incorporation processes of FOSs in metal components involve high operational costs since the equipment used for processing the metal is quite expensive, and it is important to reduce these costs.

When referring to piezoelectric-embedded sensors, there are several challenges that are associated with their implementation. For smart composite structures, it is necessary to ensure electrical insulation, electrical shielding, or electromagnetic compatibility, as piezoelectric sensors are susceptible to electromagnetic interferences. Regarding the effect of temperature, it is essential to ensure thermal coupling, as piezoelectric sensors lose their piezoelectric properties when the curing temperatures of the composites exceed the Curie temperature. In addition, the use of certain piezoelectric sensors leads to geometric disturbances, such as stress concentration at the sensor’s location and in its surrounding area, so it is necessary to optimize the sensors such that they are small and light. Finally, the use of embedded piezoelectric sensors requires a large, complicated, and power-consuming monitoring system since each sensor requires a monitoring channel and an adequate number of wires to be connected. However, technological progress has been emerging to manufacture nanoscale wires via printing or chemical deposition to decrease the quantity of wire used [137].

Regarding applications in metallic materials’ structures, the challenges related to the characteristics of embedded piezoelectric sensors are identical to those related when applied to composite structures. However, concerning the embedding process of sensors, these are already distinct, since, in this segment, the processes are thus far mainly based on additive or solid-state-manufacturing processes. Therefore, some challenges to be overcome in the future are the high costs associated with the equipment for the metal processing, which then extend to other processing technologies, e.g., processes that use fusion of the base material.

FOSs and PSs are currently the main technologies used for the incorporation of ESs into structural components, although the micro- and nanotechnology fields have shown interesting results with respect to ensuring the possibility of implementing sensory networks in variable structures and topologies. As a consequence, more sensors will generate more monitoring data, requiring the development of more efficient models for data analysis and processing [138,139]. In addition to FOSs and PSs, there are also other technologies, such as capacitive methods and electromagnetic techniques (for example, eddy currents), and materials with characteristics and properties that can be used for structural monitoring, such as shape memory alloys. These technologies generally use thin-film sensors, microstrips, or nanotubes; therefore, problems may occur related to fixing thin-film or electromagnetic interferences in microstrips.

According to the authors of this work, there are certainly numerous challenges to solve when it comes to embedding different types of sensors into structural components. However, one of the primary solutions to many of the challenges presented is the possibility of implementing hybrid systems. Hybrid systems with FOSs and PSs, for example, as presented by Yu et al. [140], provide synergy for these types of applications.

Structures’ durability with respect to embedded sensors is a main concern, so researchers have also investigated the ESs with respect to determining their effects on the mechanical behavior of a host structure. Warkentin and Crawley et al. [141] tested graphite/epoxy coupons with embedded integrated circuits on silicon chips, showing a 15% decrease in the ultimate strength of the host laminate with the embedded chips. In addition, Crawley et al. found that the ultimate strength of a graphite/epoxy laminate was reduced by 20% when a piezoceramic was embedded in the composite. Chow et al. [142] performed an analytical study that showed interlaminar stresses were five times higher with the embedment of an inert, rectangular implant in a graphite/epoxy laminate. They indicated the integrity of smart structures was affected due to the insertion of sensors/actuators [143].

With regard to FOSs’ incorporation, the research indicates that there was no degradation in the compressive strength when the optical fibers were placed parallel to reinforcing fibers, and there was no change in mechanical behavior due to embedded optical fibers [72,144,145].

The integration of sensors inside composite and metallic parts is still in the early stages of development, mainly for metal components. Thus, there is scarce literature available to compare their performance, either structurally or in terms of efficiency and economy. Nevertheless, given the trends in new reinforcement techniques, combined with the potential for digital fabrication, it is possible to conclude that there is potential in the incorporation of sensors inside components without compromising the structural integrity of the components.

Monitoring metallic components is significantly more difficult than monitoring polymeric components when using the ESs presented due to the sensor incorporation process. The incorporation of instrumentation or electronic sensors into structural components can sometimes endanger the component’s integrity and lead to problems associated with sensor fixation before embedment because glues and adhesives can be degraded under certain circumstances. Thus, according to the authors of this work, the application of metallic components manufactured from multifunctional materials and high-sensorial property materials will be the future of SHM.

SHM risk analysis is of the upmost value for companies such as insurance companies, and the use of computational methods are gaining significant relevance. Chang et al. [146] explored the feasibility of integrating built-in piezoelectric-based diagnostic techniques with a progressive failure analysis to monitor damage in composite structures. Saravanos et al. [147] developed a coupled analysis of a layered composite structure with embedded piezoelectric sensors and actuators. Giurgiutiu et al. [37] investigated the use of finite element analysis to simulate various SHM methods with piezoelectric wafer sensors. A physics-based model incorporating PZT sensor measurement was developed by Ghoshal et al. [148] to study acoustic wave generation and propagation in plates. Kim et al. [149] developed a finite element-based methodology to model embedded sensors in delaminated composite structures with piezoelectric sensors. Their results showed that embedded sensors provide more information on delamination than surface-mounted systems. The strength of PZT piezoelectric sensors is usually significantly lower than that of their host structures. Yan et al. [150] developed a method for the online detection of cracks in composite plates with embedded piezoelectric sensors using wavelet analysis. Butler et al. [151] investigated computational models focusing on PZT sensors, actuators, and associated techniques for damage detection.

The capability and versatility of the mechanics model with the coupled quasi-static and free dynamic response of composite functionality in an active (applied voltages) or sensory (applied force/displacement) mode were critical for assessing risk analysis-focused SHM. However, the computational methods for evaluating embedded sensors in metallic parts are limited; thus, researchers must focus on this area due to the extensive use of metal parts in SHM.

## 7. Conclusions

Embedded sensors currently represent one of the main fields of sensing technology; therefore, the scientific community has focused its efforts on the development and optimization of a set of technologies that ensure the continuous monitoring of structural integrity. SHM systems use a vast range of techniques; however, Fibre-Optic Sensors (FOSs) and Piezoelectric Sensors (PSs) have proven that, through the right technological processes, ESs can be incorporated into components or structures.

The selection of smart sensors or the technology underlying them is fundamental to the type of monitoring that is intended to be performed, i.e., each embedded sensor is developed and optimised to monitor certain physical and mechanical properties in specific structures and perform under specific conditions. Regardless of the type of embedded sensors or smart-sensing technology, there are limitations of use related to the physical, chemical, and mechanical limits of each. In this sense, with the correct selection of embedded sensors and technological process for its integration, it is possible to obtain structures or structural components that are reliable, attaining the possibility of continuous monitoring is both effective and accurate.

The review of studies developed on embedded sensors in structural components showed that over the last 15 years, there has been exponential growth not only in terms of the technological progress but also in the development of new applications that use composite materials, essentially promoted by their increasing use in industrial applications. However, the development of applications with metallic components has suffered few advances, evidencing their scarce and barely industrialised nature, so it is crucial to allocate resources to boost the development of smart metallic systems.

## Figures and Tables

**Table 1:** Types of optic fibre sensors (adapted from [30,56]).

	Point Sensor	Quasi-Distributed Sensor	Distributed Sensor
**Sensors**	Fabry–Perot Cavity Fibre Bragg Grating Long gage sensor	Fibre Bragg Grating	Raman/Rayleigh Brillouin
**Measurands**	Strain (displacement, pressure, temperature)	Strain (displacement, acceleration, pressure, relative fissure, inclination, etc.)	Temperature/Strain
**Modulation Method**	Phase-modulated optical fibre sensors, or interferometers	Wavelength	Intensity
**Schematic**	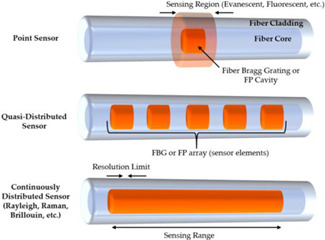		

**Table 2:** Overview of applications and methodology of integrating Fibre Bragg grating (FBG) Sensors for composite structural components.

Author	Methodology of Integrating Sensors	Measurements	Sensitivity	Applications
Kuang et al. [62] (2001)	Open Contact Moulding Processes	Strain	-	Carbon Fibre/Epoxy Laminate.
Keulen et al. [63] (2011)	Open Contact Moulding Processes	Strain	0.001 nm/mε	Composite Panel
Ramly et al. [64] (2012)	Resin Infusion Processes.	Strain	-	Sandwich Composite Panel
Bremer et al. [65] (2017)	Open Contact Moulding Processes	Strain and Crack	0.0033 mm/N	
Oromiehie et al. [66] (2018)	Automated Fibre Placement	Defects	-	Composite Components for the Aerospace Industry
Kousiatza et al. [67] (2019)	Fused Filament Fabrication.	Residual Strain	-	Complex Lightweight Structures
Mieloszyk et al. [68] (2021)	Open Contact Moulding Processes	Temperature and Strain	-	Marine Applications
Hurtado et al. [69] (2021)	Resin Transfer Moulding	Strain	up to 7500 μ	Fibre-Reinforced Polymer Structure Failure

**Table 3:** Overview of applications and methodology of integrating both Fibre Bragg grating (FBG) Sensors and Extrinsic Fabry–Perot Interferometers (EFPI) for composite structural components.

Author	Methodology of Integrating Sensors	Measurements	Sensitivity	Applications
Leng et al [71] (2003)	Open Contact Moulding Processes	Strain	-	Carbon Fibre-reinforced Polymer
Oliveira et al. [70] (2008)	Compression Moulding Processes	Strain	2.6 με/N	Carbon Fibre-reinforced Polymer

**Table 4:** Overview of applications and methodology of integrating Fibre Bragg grating sensors in metal structural components.

Author	Methodology of Integrating Sensors	Measurements	Sensitivity	Applications
Li et al. [73] (2000)	Magnetron Sputtering and Electroplating	Temperature	0.0245 nm/°C	Nickel and Stainless-Steel Structures.
Li et al. [74] (2001)	Magnetron Sputtering and Electroplating	Temperature	0.021 nm/°C	Nickel and Stainless-Steel Structures
Li et al. [75] (2003)	Magnetron Sputtering and Electroplating	Strain Temperature	$1.245\times 10^{-3}$ nm/με 0.0334 nm/°C	Monitoring the Accumulation of Residual Strain
Li et al. [76] (2004)	Layered Manufacturing	Temperature	-	Turbine Blades and others’ Rotary Metal Tooling
Alemohammad et al. [77] (2011)	Magnetron Sputtering and Electroplating	Residual Stress Temperature	21 pm/°C.	Metal Cutting Tools
Schomer et al. [78] (2017)	Ultrasonic Additive Manufacturing	Temperature	-	High-Temperature Environments
Grandal et al. [79] (2018)	Laser Cladding Technology	Strain Temperature	29 pm/°C–23 pm/°C. 0.9 pm/με–1 pm/με.	High-Temperature Environments
Jinachandran et al. [80] (2018)	Metal Packaging using Stainless Steel and Tin	Strain Temperature	0.4456 με/N 11.16 pm/°C	Iron Pipelines and other Ferromagnetic Components
Chilelli et al. [81] (2019)	Ultrasonic Additive Manufacturing	Cracks	Length of 0.286 ± 0.033 mm	Complex Systems
Hehr et al. [82] (2020)	Ultrasonic Additive Manufacturing	Residual Stress Temperature Delamination	-	Fibre-Routing Designs and Alloy Systems

**Table 5:** Overview of applications and methodology of integrating Lead Zirconate Titanate (PZT) piezoelectric sensors into composite structural components.

Author	Methodology of Integrating Sensors	Measurements	Sensitivity	Applications
Wu et al. [88] (2006)	Mounted on Reinforced Concrete	Damage	1 to $15\times 10^{-3}$ V	Reinforced Concrete Structures
Konka et al. [89] (2011)	Open-Contact Moulding Processes	Stress Ultimate Strength	-	Composite Structures
Tang et al. [94] (2011)	Vacuum-Assisted Resin Transfer Moulding	Failure	-	Damage Prediction in Composites
Talakokula et al. [95] (2015)	Mounted on Reinforced Concrete	Corrosion	-	Reinforced Concrete Structures
Karayannis et al. [96] (2016)	Mounted on Reinforced Concrete	Admittance Signatures	-	Concrete Beams’ Cracking
Gopalakrishnan et al. [97] (2019)	Mounted on Reinforced Concrete	Conductance Signatures	-	Reinforced Concrete Structures
Ahmadi et al. [98] (2021)	Mounted on Reinforced Concrete	Corrosion (Electro-Mechanical Impedance)	-	Reinforced Concrete Structures
Sha et al. [99] (2021)	Encapsulation with Concrete, Epoxy Resin, and Curing Agent	Stress (Electromechanical Impedance)	-	Reinforced Concrete Structures
Huijer et al. [100] (2021)	Open-Contact Moulding Processes	Degradation Failure (Acoustic Emissions)	-	Carbon Fibre-Reinforced Plastics
Gayakwad et al. [101] (2022)	Mounted on Concrete	Damage (Electromechanical Impedance)	-	Concrete Structures
Wu et al. [90] (2022)	Mounted on Reinforced Concrete	Strain	169 to 278 pC/με	Concrete Structures

**Table 6:** Overview of applications and methodology of integrating other piezoelectric sensors into composite structural components.

Author	Types of Sensors	Methodology of Integrating Sensors	Measurements	Applications
Lin et al. [91] (2001)	Thin Dielectric Film	Open-Contact Moulding Process or Others	Damage Material Degradation	Metallic and Composite Structures
Takagi et al. [92] (2006)	Piezoelectric Fibres	Open-Contact Moulding Process	Active Vibration	Carbon Fibre-Reinforced Polymer Composites
Downey et al. [93] (2017)	Carbon Nanotubes	Mounted on Concrete	Damage Failure	SHM in Civil, Mechanical, and Aerospace Structures

**Table 7:** Overview of applications and methodology of integrating different types of sensors such as Thin Dielectric Films, Lead Zirconate Titanate (PZT) Piezoelectric Sensors, Piezoelectric Ultrasonic Transducers, and Piezoelectric polyvinylidene fluoride (PVDF) in metal structural components.

Author	Methodology of Integrating Sensors	Measurements	Sensitivity	Applications
Lin et al. [91] (2001)	Open Contact-Moulding Process or Others	Damage Material Degradation	-	Metallic and Composite Structures
Hossain et al. [102] (2016)	Electron Beam Melting	Stress	0.42 to 0.53 V/kN	Pressure Tubes and Turbine Blades
Tseng et al. [103] (2018)	Casting	Temperature	0.37 ℃/bit	Solid Metal Structural Component
Altammar et al. [104] (2018)	Sandwich Panel Manufacturing	Wave Propagation Analysis (Damage)	-	Laminate Structures
Yanaseko et al. [105] (2019)	Hot-Pressing Process	Displacement	14.0 mV/μm	Evaluation of Viscosity Characteristics
Ramanathan et al. [106] (2021)	Ultrasonic Additive Manufacturing	Strain	9.4 mV/με	Functionalised Metal Structures

**Table 8:** Overview of applications, type of embedded sensors used, and methodology of integrating sensors for composite structural components.

Author	Types of Sensors	Methodology of Integrating Sensors	Measurements	Applications
Ong et al. [107] (2008)	Passive and Wireless Inductor–Capacitor Resonant Circuit	Mounted on Reinforced Concrete	Water Content	Real-Time Monitoring of Water Content in Structures
Pinto et al. [108] (2012)	Shape Memory NiTi Alloy	Open Contact Moulding Process	Strain Distribution Damage	Carbon-Reinforced plastic Composites
Sebastian et al. [109] (2014)	Glass Fibre Coated with Carbon Nanotube	Open Contact Moulding Process	Strain	Carbon-Reinforced plastic Composites
Teng et al. [110] (2019)	Microstrip Patch Antenna	Mounted on Reinforced Concrete	Moisture Content Deterioration	Reinforced Concrete Structures
Santiago et al. [111] (2020)	Capacitance System	Additive Manufacturing.	Deformation Impacts	Metal and Ceramic Lattices
Cougnom et al. [112] (2021)	Thin Films	Magnetron-Sputtering Deposition and Open Contact-Moulding Process	Heating Elements	Fabrication of Heating Elements.
Meoni et al. [113] (2021)	Carbon Nanotubes	Mounted on Reinforced Concrete	Strain	Reinforced Concrete Structures
Gino et al. [114] (2022)	PZT Powder	Resin Infusion Processes	Loads (Through the Electrical Signal)	Glass Fibre-reinforced Polymer

**Table 9:** Overview of applications, type of embedded sensors used, and methodology of integrating sensors for metal structural components.

Author	Types of Sensors	Methodology of Integrating Sensors	Measurements	Applications
Li et al. [73] (2000)	Thin-Film Thermo-Mechanical Sensor	Laser Assisted Metal Deposition	Strain	Nickel and Stainless-Steel Structures
Cheng et al. [120] (2007)	Thin-Film Thermocouple	Ultrasonic Metal Welding	Temperature	Nickel, Stainless-Steel, and Titanium Alloy Tools
Zhang et al. [121] (2008)	Micro Ring Sensor	Laser-Assisted Metal Deposition	Temperature	Monitoring of Manufacturing Processes
Hahnlen et al. [122] (2010)	Shape Memory NiTi Alloy	Ultrasonic Additive Manufacturing	Temperature	Monitoring of Manufacturing Processes
Juhasz et al. [123] (2020)	Passive Sensor-Printed	Hybrid Manufactured Metal Structure	Strain	Metal Structural Components.
Sholl et al. [124] (2021)	Eddy Current Sensors	Laser Powder Bed Fusion.	Crack Propagation	Metal Structural Components.
